# Inflorescence Development and Floral Organogenesis in *Taraxacum kok-saghyz*

**DOI:** 10.3390/plants9101258

**Published:** 2020-09-24

**Authors:** Carolina Schuchovski, Tea Meulia, Bruno Francisco Sant’Anna-Santos, Jonathan Fresnedo-Ramírez

**Affiliations:** 1 Departamento de Fitotecnia e Fitossanidade, Universidade Federal do Paraná, Rua dos Funcionários, 1540 CEP 80035-050 Curitiba, Brazil; carolina.sschu@gmail.com; 2 Molecular and Cellular Imaging Center, The Ohio State University, 1680 Madison Avenue, Wooster, OH 44691, USA; meulia.1@osu.edu; 3 Laboratório de Anatomia e Biomecânica Vegetal, Departamento de Botânica, Setor de Ciências Biológicas, Universidade Federal do Paraná, Avenida Coronel Francisco H. dos Santos, 100, Centro Politécnico, Jardim das Américas, C.P. 19031, 81531-980 Curitiba, Brazil; brunofrancisco@ufpr.br; 4 Department of Horticulture and Crop Science, The Ohio State University, 1680 Madison Avenue, Wooster, OH 44691, USA

**Keywords:** Asteraceae, flower morphology, neo-domestication, rubber dandelion, scanning electron microscopy (SEM), tribe Cichorieae

## Abstract

Rubber dandelion (*Taraxacum kok-saghyz* Rodin; TK) has received attention for its natural rubber content as a strategic biomaterial, and a promising, sustainable, and renewable alternative to synthetic rubber from fossil carbon sources. Extensive research on the domestication and rubber content of TK has demonstrated TK’s potential in industrial applications as a relevant natural rubber and latex-producing alternative crop. However, many aspects of its biology have been neglected in published studies. For example, floral development is still poorly characterized. TK inflorescences were studied by scanning electron microscopy. Nine stages of early inflorescence development are proposed, and floral micromorphology is detailed. Individual flower primordia development starts at the periphery and proceeds centripetally in the newly-formed inflorescence meristem. Floral organogenesis begins in the outermost flowers of the capitulum, with corolla ring and androecium formation. Following, pappus primordium—forming a ring around the base of the corolla tube—and gynoecium are observed. The transition from vegetative to inflorescence meristem was observed 21 days after germination. This description of inflorescence and flower development in TK sheds light on the complex process of flowering, pollination, and reproduction. This study will be useful for genetics, breeding, systematics, and development of agronomical practices for this new rubber-producing crop.

## 1. Introduction

The genus *Taraxacum* Wigg. (dandelion) belongs to the family Asteraceae, subfamily Cichorioideae, tribe Cichorieae, and sub-tribe Crepidinae [1]. Asteraceae (Compositae) is a large family of angiosperms. Its members have an inflorescence called a capitulum which is characterized by flowers organized on a receptacle and bracts (phyllaries) forming an involucre [2]. The tribe Cichorieae includes annual and perennial herbs that produce latex, i.e., they are characterized by the presence of lactiferous canals in subterranean and aerial parts [1].

Members of the *Taraxacum* genus are commonly found throughout the temperate region. These plants form a polyploid complex with both sexual and apomictic modes of reproduction [3,4,5]. *Taraxacum kok-saghyz* (TK), unlike many dandelion species, is diploid (2n = 2x = 16), with self-incompatible sexual reproduction [6,7,8]. While it is an herbaceous perennial dandelion, TK can be grown in annual or biennial cropping systems. Each plant produces many capitula supported by separate peduncles. The capitula contain clusters of flowers surrounded by a rosette of involucral bracts with small pointed edges [9].

*Taraxacum kok-saghyz* has been studied as an important alternative source of natural rubber, cis-1, 4-polyisopropene [10,11,12,13,14], a raw material that can be used for producing both industrial and medical products. TK rubber is especially valuable because it is renewable and of superior quality, presenting a uniformly high molecular weight polymer when compared with synthetic rubber [15]. The Para rubber tree (*Hevea brasiliensis*) is currently the primary source of natural rubber. However, there are several reasons to seek alternative sources including *Hevea* rubber’s potential to cause allergic reactions and the threat of the future decline in natural rubber production caused by a fungal disease (South American Leaf Blight) ravaging *Hevea* plantations. Additionally, competition for land use by other crops and an increase in demand for natural rubber can create latex shortages [7,16,17]. Neo-domestication of alternative latex crops would enable the development of this sustainable industry and reduce the dependence on fossil fuel sources to produce synthetic substitutes [7,10,17]. TK is a promising alternative as a next-generation natural rubber resource [18,19]. It has ideal agronomical properties for efficient production as an annual crop including fast development and high biomass production [17]. Capitalizing on research studies in TK domestication and rubber content extraction and analyses [11,12,13,14,16,17,20], a few companies have invested in the use of TK as an alternative rubber-producing crop [19]. Some studies also indicate that TK has the potential to be a source of additional important products, including inulin and proteins [14,21].

There is an ongoing breeding effort to fully domesticate TK in order to improve yield and rubber concentration [22,23,24,25] and to introduce desirable agronomic traits. Some techniques have been developed to accelerate breeding in TK, such as CRISPR/Cas9-induced genome editing [26]; polyploid induction using colchicine [27]; the development of simple-sequence repeat (SSR) markers from available expressed-sequence tag (EST) data to study genetic diversity in TK populations [28]; the use of RNA sequencing to generate a transcriptome database for TK roots [25]; studies of trait variance, inter-trait correlation, and heritability for traits related to rubber yield [24]; and genetic transformation mediated by *Agrobacterium* [29]. However, additional agronomic studies are needed for TK to become a commercial crop [30], particularly intensive breeding using mass selection and crossing [6]. The understanding of flower induction and floral development is fundamental to improve success in crossing and seed production and to further improve the TK germplasm [31]. Additionally, the flowering habit is related to rubber yield. Higher rubber yields were observed in winter-type plants, i.e., plants that grow vegetatively and need a cold/vernalization period before flowering, compared to spring-type (early-flowering) plants. Therefore, studies of the flowering process will be useful for breeding rubber-enriched germplasm [31,32,33].

Despite its importance, floral development has not been previously characterized in TK. To better understand the transition from vegetative to reproductive growth, the objective of this study is to describe the developmental stages of the early inflorescence (capitulum) in TK, including floral organogenesis, and their correlation to the morphological stages observed in TK plants growing under controlled conditions.

## 2. Results

### 2.1. Morphological Aspects

**Figure 1:**
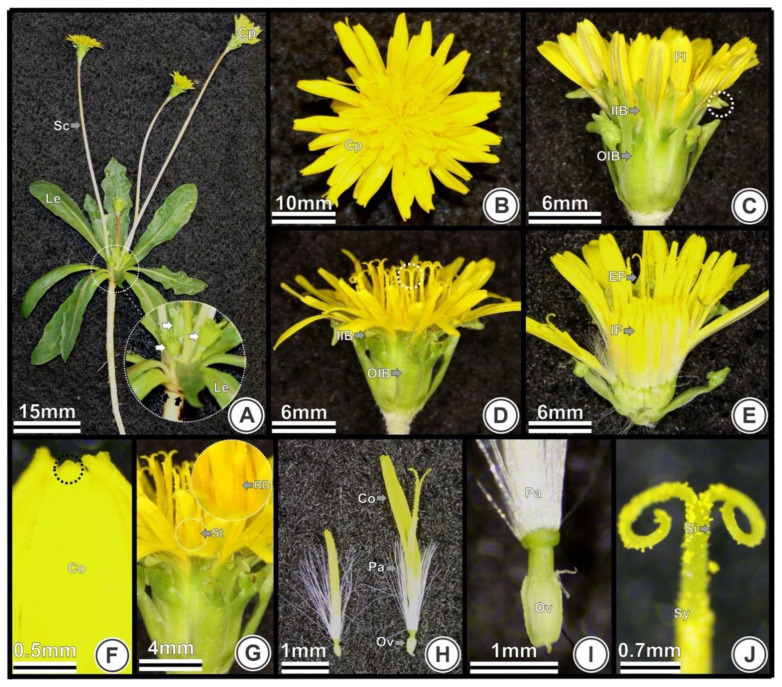
Flower and inflorescence morphology in *Taraxacum kok-saghyz*. Different views of the inflorescences (**A**–**E**) and flowers (**F**–**J**) under digital camera (**A**,**B**) and stereomicroscope (**C**–**J**). (**A**) Plant with rosette leaves and solitary capitulum on each scape. Newly formed inflorescences (white arrow) and leaves insertion (black arrow) showed in detail; (**B**) Top view: homogamous capitulum; (**C**) Lateral view: flowers protected by the inner and outer involucral bracts, pointed edges on inner involucral bracts (white circle); (**D**) Lateral view: styles and stigmas (white circle) and involucral bracts; (**E**) Longitudinal section: inner flowers closed and external flowers opened; (**F**) Lateral view: toothed corolla (black circle); (**G**) Epipetalous stamens with rimose dehiscence (in detail); (**H**) Lateral view: corolla, pappus ring and inferior ovary; (**I**) Detail of the ovary and pappus; (**J**) Lateral view of a bifid style and stigma. Abbreviations: Co, corolla; Cp, capitulum; Fl, flowers; IIB, inner series of involucral bracts; Le, leaves; Ov, ovary; OIB, outer series of involucral bracts; Pa, pappus; RD, rimose dehiscence; Sc. scapes; Si, stigma; St, stamens; Sy, style.

**Figure 2:**
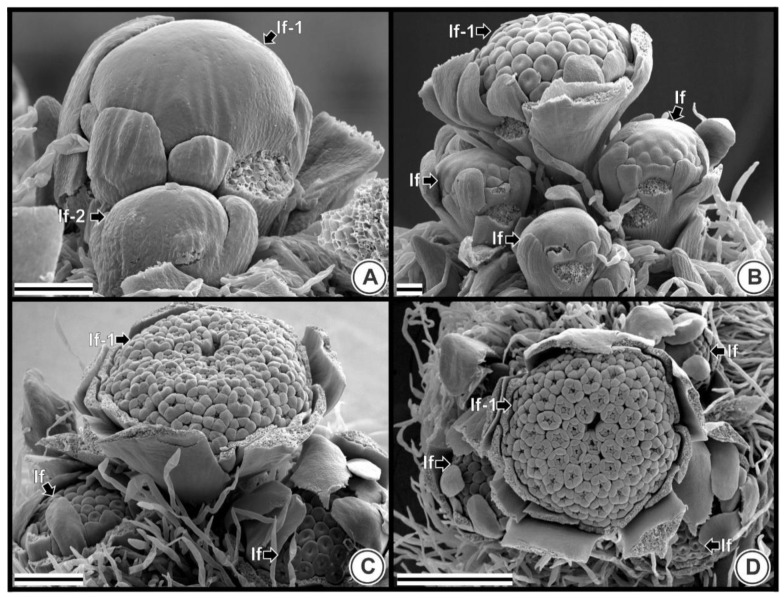
Inflorescences arrangement in the plant of *Taraxacum kok-saghyz*. Pictures were obtained by scanning electron microscopy; (**A**) Lateral view of a set of inflorescences. Inflorescence 1 in the back and a smaller inflorescence 2 in front (21 days after germination, DAG); (**B**) Lateral view of four different stages of inflorescences at 24 DAG; (**C**) Three different stages of inflorescences are observed in this picture, 30 DAG; (**D**) Top view of four inflorescences at 30 DAG. Abbreviations: DAG, days after germination; If, inflorescence. Scale bars = 150 µM.

**Figure 3:**
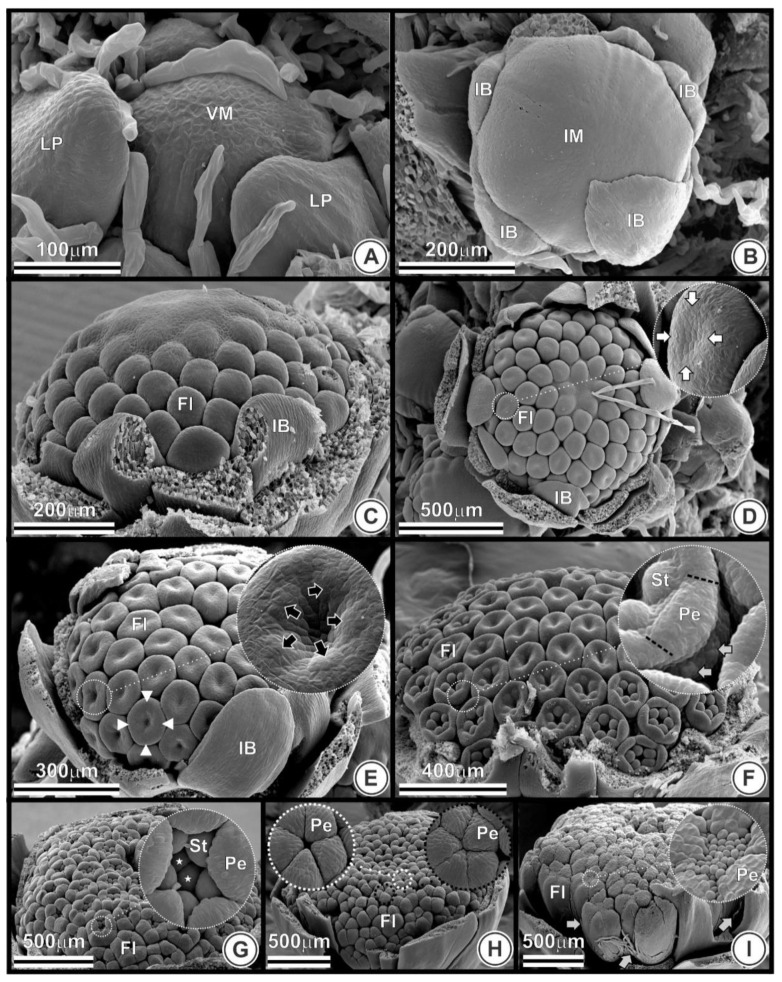
Inflorescence developmental stages in *Taraxacum kok-saghyz*. Each image shows one of the nine stages (scanning electron microscopy from 5 to 33 days after germination) and morphological aspects of the peripheral flowers in the inflorescence; (**A**) Vegetative meristem, dome-shaped and surrounded by leaf primordial; (**B**) Inflorescence meristem with flattened and widened aspect, involucral bracts, and no sign of flower primordium; (**C**) Flowers arising in an acropetal order; (**D**) Central concavity (white arrows); (**E**) Corolla ring (white arrow heads) and stamens primordia (indicated by black arrows in the detail); (**F**) Petals and five stamens more visible and pappus primordia (gray arrows in the detail). The petals boundaries are showed in the detail (black lines); (**G**) Stamens, carpel primordia (stars) and petals enlarging and touching each other; (**H**) Petals almost (white circle) and fully (black circle) covering the stamens; (**I**) Petals completely touching each other, longer than in previous stages, and pappus (gray arrows). Abbreviations: Co, corolla; CR, corolla ring; Fl, flowers; IB, involucral bracts; IM, inflorescence meristem; LP, leaf primordia; Pa, pappus; Pe, petals; St, stamens; VM, vegetative meristem.

**Figure 4:**
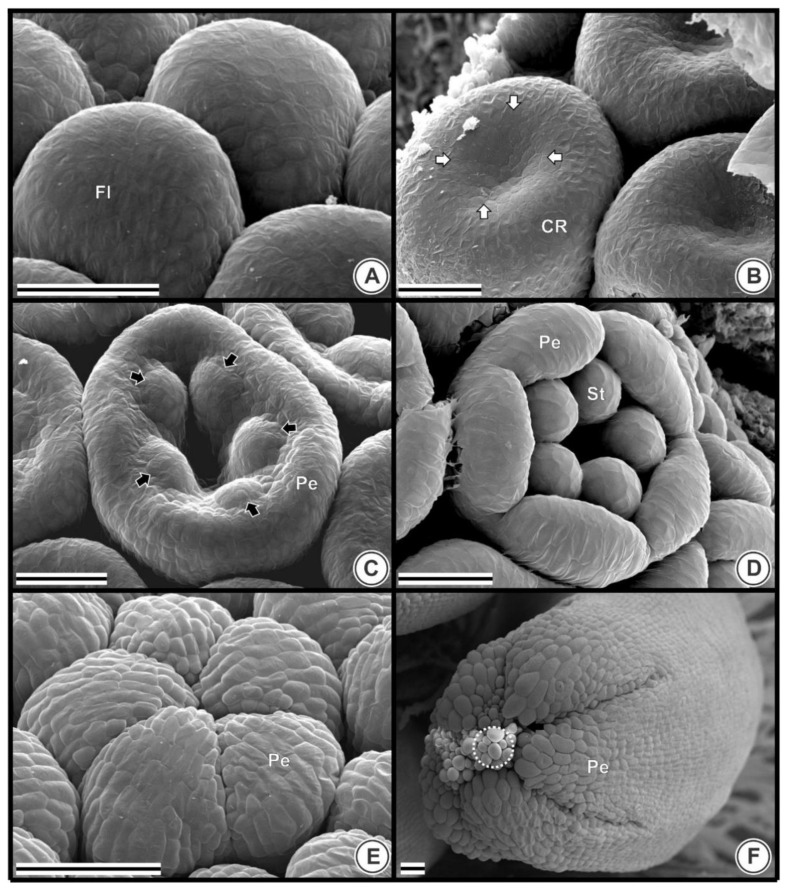
Flower organogenesis in *Taraxacum kok-saghyz* (scanning electron microscopy from 5 to 33 days after germination). (**A**) Round-shaped; (**B**) Center concavity (white arrow) and corolla ring (CR) primordium; (**C**) Petals and stamens primordia (black arrows); (**D**) Enlarged petals and stamens; (**E**) Petals enclosing the androecium: corolla lobes touching each other; (**F**) Corolla lobes bend towards the floral apex, approach each other until they meet at their margins where they interlock by epidermal cells (white circle). Scale bars: 50 μm. Abbreviations: Co, corolla; CR, corolla ring, Fl, flowers; Pe, petals; St, stamens.

*Taraxacum kok-saghyz* is an herbaceous plant with leaves forming a rosette (Figure 1A) and produces small flowers that are tightly packed, forming a head-like inflorescence called capitulum (Figure 1A). The inflorescence-stems (scapes) are leafless, with one solitary capitulum at each apex (Figure 1A). The inflorescence emergence occurs near the point of the leaf’s insertion into the stem (Figure 1A). Flowers are all alike (homogamous capitulum; Figure 1B), with the same corolla shape and sexual configuration (bisexual; Figure 2, Figure 3 and Figure 4). The flowers are surrounded by two layers of involucral bracts (phyllaries; Figure 1C,D). The outer series of bracts show a lanceolate and pointed horn at the edge. The inner series of bracts show a lanceolate erect edge (Figure 1C). These bracts act like sepals and protect the flowers during development. Each capitulum contains dozens of small yellow ligulate flowers that develop from the outside to the inside region of the capitulum (Figure 1D,E). Flowers have a five-toothed ligule (Figure 1F) and five epipetalous stamens with rimose dehiscence (Figure 1G, Figure 3 and Figure 4). The calyx is represented by a circle of unbranched hairs (pappus) forming a ring surrounding the corolla, which is associated with fruit dispersion (Figure 1H,I). The pappus and the corolla are inserted above the inferior ovary (Figure 1H,I) with two carpels (Figure 3). The style is bifid (Figure 1J).

In TK plants, successive inflorescences emerge in a spiral distribution surrounding the first inflorescence (Figure 2) in a basipetal order. Therefore, it is possible to distinguish inflorescences at different developmental stages in the same plant (Figure 1 and Figure 2). The first formed capitulum does not show flower primordia arising, even with the second inflorescence already in development (Figure 2A). In Figure 2B, the first formed inflorescence already shows flower primordia and initial floral organogenesis, whereas the other surrounding younger inflorescences are still beginning to form flower primordia or are even in the stage where no flower primordia are visible. In Figure 2C,D it is possible to observe more developed stages of the inflorescences and the first formed inflorescence formed shows a more advanced stage.

### 2.2. Inflorescence Developmental Stages

Nine stages of TK inflorescence (capitulum) development (A–I), from vegetative to reproductive meristem, were identified and detailed in this study (Figure 3). Stage A (Figure 3A) refers to the vegetative meristem (shoot apical meristem), which is dome-shaped and surrounded by leaf primordia.

Stage B represents the conversion from vegetative to reproductive meristem (Figure 3B), where a transition phase is recognizable and first observed in this study at 21 days after germination (DAG) (Figure S1), representing the beginning of the inflorescence meristem formation. In stage B, the shoot apical meristem dome begins broadening and flattening, and the involucral bract primordia along the periphery (phyllaries) are formed (Figure 3B). At this stage, there is no sign of flower primordia along the inflorescence meristem.

In stage C (Figure 3C), the flower primordia begin to emerge in an acropetal (centripetal) order in the inflorescence meristem. For a better view of this floral stage, the involucral bracts have been removed in stages C to I (Figure 3C–I).

All flowers are visible in stage D (Figure 3D), completing the remaining uncommitted meristematic region of the inflorescence. At stage D, the peripheral flowers start the organogenesis of the corolla whorl, showing the beginning of concavity in the center of the flower.

At stage E (Figure 3E), all flowers show the center concavity. Organogenesis development continues and the peripheral flowers present corolla ring and early androecium.

At stage F (Figure 3F), organogenesis proceeds acropetally on the capitulum, and the peripheral flowers show more visible development of five petals and five stamens. The central flowers in the capitulum show the early formation of the corolla and androecium whorls.

Stage G (Figure 3G) shows inflorescence, with the peripheral flowers having corolla lobes enlarging and touching each other.

At stage H (Figure 3H), all the flowers have elongated petals, covering the stamen. During stage I (Figure 3I), flowers are closed, longer than in previous stages, and numerous pappus members are visible.

### 2.3. Floral Organogenesis

When the flower arises in the inflorescence meristem, it has a convex-round shape (Figure 4A). The first sign of whorls is seen in Figure 4B, where the flower center becomes depressed, forming a concavity, and the corolla ring primordium is distinguishable. Next, corolla lobes start emerging at the border of the flower, the center concavity increases, and five stamen primordia appear in the center alternately to the five petal primordia (Figure 4C). It is notable that all five corolla lobes develop simultaneously in a flower. Similarly, all five stamens are developed simultaneously in a single flower. At this stage, pappus is initiated (shown in Figure 3F).

Subsequently, petals and stamens are clearly distinct, corolla lobes become enlarged, and stamens become round-shaped on the top (Figure 4D). Subsequently, corolla lobes elongate, with their edges touching each other (Figure 4E). Afterward, the corolla lobes bend towards the floral apex and approach each other until they meet at their margins, where they interlock by epidermal cells (Figure 4F). Finally, corolla lobes fuse postgenitally and the congenitally fused portion of the tube elongates (Figure 4F).

### 2.4. Timing of Inflorescence and Floral Organogenesis Correlated to Plant Development

All nine inflorescence stages of development were observed and correlated to the age of the plants growing in the greenhouse (in DAG; Figure S1). At 5, 10, 15 and 18 DAG, the observed plants showed only vegetative meristems. However, at 21 DAG, the transition to reproductive/inflorescence meristem (stage B) and the initiation of flowers in some inflorescence meristems (stage C) were first observed in some plants. Stages D to H were first observed in plants at 24 DAG. Finally, stage I only appears at 30 DAG. Stage A (vegetative meristem) appears on each sampling date (Figure S1).

**Figure 5:**
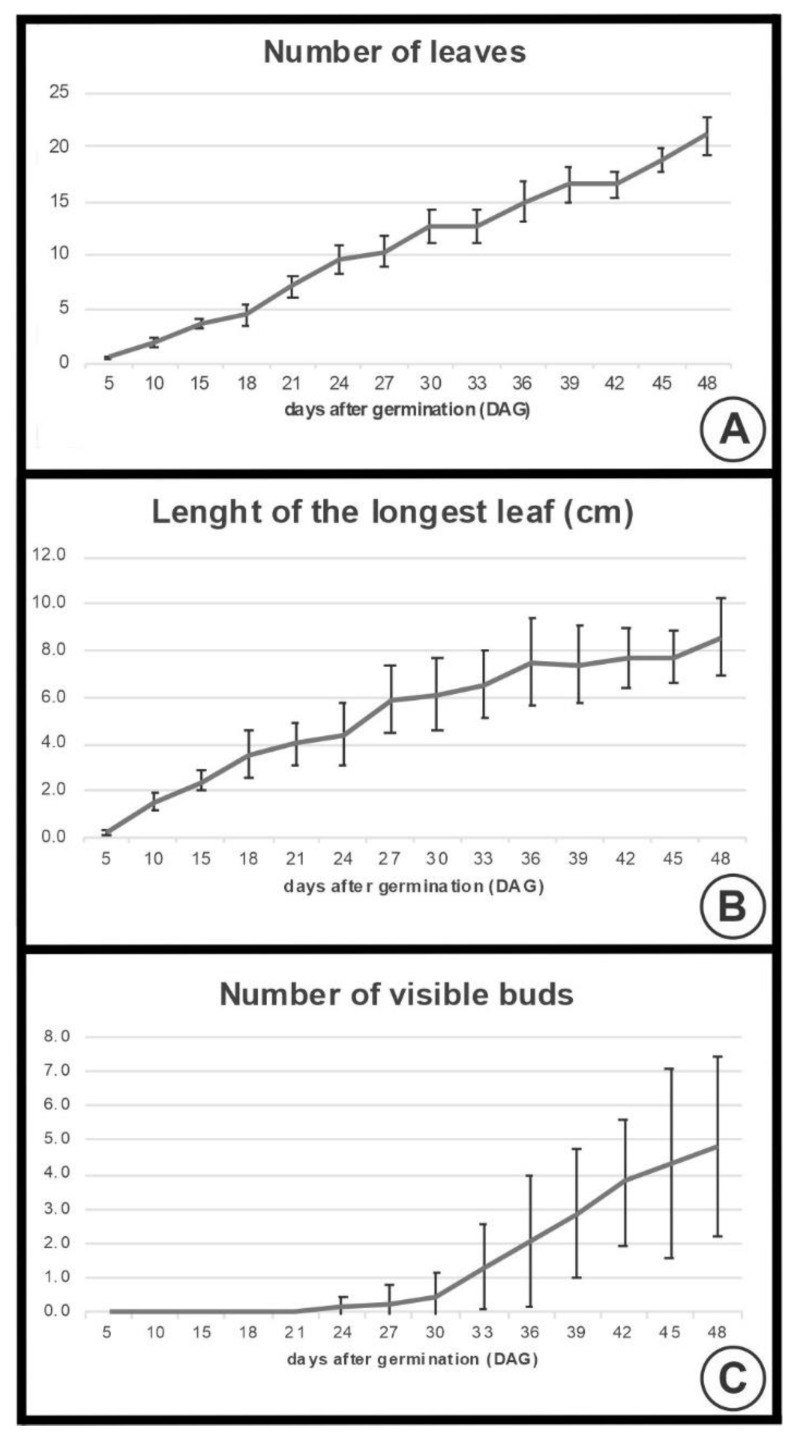
Development of Taraxacum kok-saghyz 5 to 48 days after germination (DAG). (**A**) Number of leaves. (**B**) Length of the longest leaf (cm). (**C**) Number of visible inflorescence buds. *n* = 13–31. Bars represent the standard deviation (SD).

The first trait visually analyzed was the number of leaves (Figure 5A). At 5 DAG, plants showed a mean of 0.6 leaves, while 7.2 leaves were visible at 21 DAG when some plants began the transition from vegetative to reproductive meristem (Stage B). Finally, at 48 DAG, 21.1 leaves were visible.

The results regarding the longest leaf measurements are shown in Figure 5B. The development of leaves was observed at 5 DAG, with the longest leaf measuring 0.2 cm. The longest leaf was 4.0 cm when the transition to reproductive meristem occurred (21 DAG), and finally reached a mean of 8.5 cm at 48 DAG.

The number of visible inflorescence buds in each of the DAG can be observed in Figure 5C. Until 21 DAG, no buds are externally visible. At 24 DAG, a mean of 0.1 buds are visible, while 4.8 buds are visible at 48 DAG.

The overall development of plants in the greenhouse, including the number of leaves, length of the longest leaf, and number of visible buds for all DAG when plants were collected, are found in Figure S2.

### 2.5. Inflorescence Bud Appearance and Anthesis

The first inflorescence bud externally visible in the center of the plant appeared on average at 32.2 DAG. However, it was noted that the first inflorescence bud appeared in some plants as early as at 24 DAG (Figure S3). The appearance of further inflorescence buds occurred in a spiral, basipetally. The second inflorescence bud appeared at 37.6 DAG, the third bud at 38.4 DAG, and the fourth bud at 41.1 DAG. Finally, the timing of anthesis was observed to be 49.2 DAG. However, anthesis was first observed in some plants at 42 DAG, while other plants showed no inflorescence bud until 60 DAG. There was a mean interval of 17 days from the first externally visible bud to anthesis.

**Figure 6:**
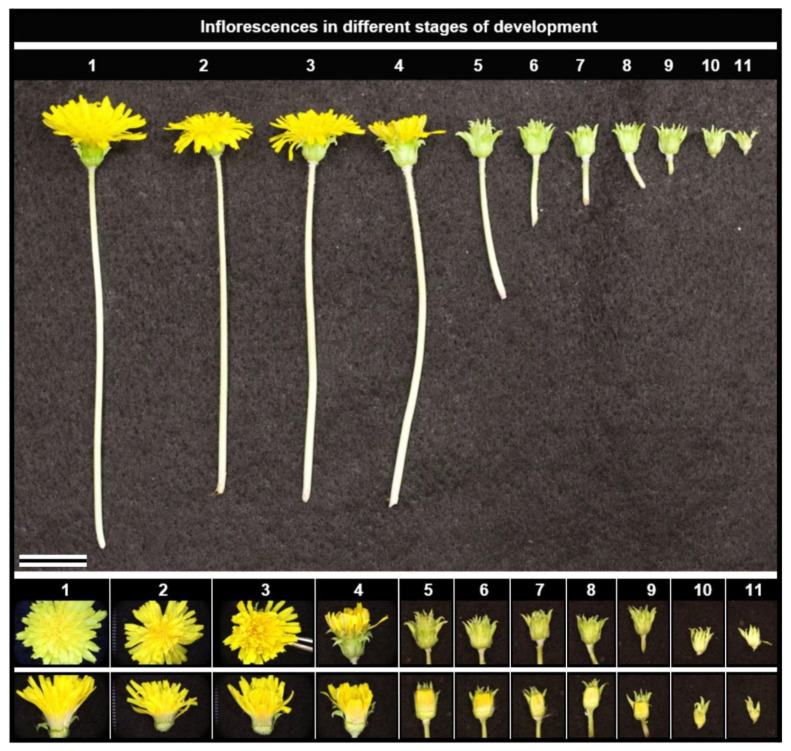
Different stages of morphological inflorescence development in *Taraxacum kok-saghyz*, showing inflorescences 1 to 11 in the same plant. The 20 mm scale bar represents the size in all the figures.

The external morphology of inflorescence development can be observed in Figure 6, where inflorescences are numbered in the order of their initiation (from 1 to 11) and collected from the same plant at 53 DAG. The first inflorescence bud was visible at 30 DAG in this particular plant. The inflorescence buds numbered 2, 3, and 4 appeared together at 35 DAG. The inflorescence bud number 5 appeared at 42 DAG, while the bud number 6 appeared at 46 DAG.

Observation of one TK plant revealed that when the first inflorescence reaches anthesis, several (as many as 6) inflorescence buds were already externally visible. Besides the visible buds, there were additional inflorescence buds in the center of the basal rosette that were smaller in size and did not appear externally (Figure S2).

## 3. Discussion

This study describes the transition from vegetative shoot apical meristem to reproductive meristem in TK plants growing under controlled conditions. The study of floral development explores a key step in inflorescence and floral initiation, the transition from vegetative meristems to inflorescence and floral meristems [34]. Our results provide a description of inflorescence development based on SEM images in TK. Nine stages of inflorescence development are proposed in this research, starting with the vegetative meristem, the conversion process to the reproductive stage, and further development of the inflorescence meristem, showing flowers initiation and organ formation in the capitulum.

Our data show that TK presents a homogamous capitulum composed of ligulate flowers in accordance with the Cichorieae tribe characteristics [2]. This structure contrasts with the frequent arrangement of the Asteraceae capitulum represented by a peripheral ring of attractive ray flowers encircling a compact cluster of disc flowers [35], as seen in sunflower [36,37] and *Gerbera* [37,38].

The transition of vegetative to inflorescence meristem (transition process) was observed here in plants at the age of 21 DAG, showing 7.2 leaves, the longest leaf measuring 4.0 cm, and 0.1 buds appearing externally in the center. Asteraceae has marked characteristics regarding the transition of vegetative meristem to inflorescence meristem. The vegetative meristematic apex is small and domed. During transition, the meristem enlarges, becoming broader and flatter [39]. Unlike observations in TK, the transition process in *Xeranthemum squarrosum* occurs with no sign of flower primordia [40].

After the transition process, the inflorescence developmental stages occurred in an acropetal order in TK. These findings are consistent with most of the Asteraceae plants [39], especially those with homogamous capitulum such as *Stilpnolepis centiflora* [41]. This acropetal sequence can also occur within some heterogamous capitula, as described in *Gerbera* [42,43]. Conversely, most species with heterogamous capitulum [e.g., *Osteospermum ecklonis* [44], *X. squarrosum* [40], *Senecio vernalis* [45], *Chrysanthemum lavandulifolium*, and *Ajania achilleoides* [41] have an acropetal order within the disk flowers, while the ray flowers (in the periphery of the capitulum) lag behind in development.

The single-born capitulum on scapes described here for TK, although present in some species of the Cichorieae tribe, is not a common feature in the tribe [1]. However, it is also described in other Asteraceae such as *Gerbera hybrida* and *Helianthus annuus* [37]. The capitulum usually presents involucral bracts (phyllaries) with the function of sepals, protecting the developing capitulum [40,46]. In TK, these involucral bracts were described and began to appear at stage B in the transition phase, forming a closed protective barrier.

The formation of pappus in Asteraceae could follow one of three developmental paths: sequential (many bristle-like pappus initiate in five points alternate to the petals), random process (pappus member primordia appear wherever space is available), and the appearance of a pappus ring [39]. In the present study, the initiation of numerous pappus primordia, forming a ring around the flower was observed for TK. The same observations were made in *S. vernalis*, where a great number of pappus bristles are formed arranged in a ring meristem [45]. The pappus formation is an important feature related to dispersal or defense against herbivory [44,47]. In *Gerbera*, the pappus bristles are modified sepals that aid dispersal [42]. In Asteraceae, sepals lost their protective function (now exerted by bracts) and became narrower and more numerous, being modified into pappus [48].

The floral organogenesis in TK is described in the present work, showing the formation of the corolla ring whorl followed by formation of the androecium. This is similar to what was reported in the common dandelion flowers [49]. This study also observed pappus primordia initiation just prior to gynoecium development. Additionally, during whorl initiation, organs in the same whorl developed simultaneously. These descriptions are in accordance with most Asteraceae species [39]. However, organ initiation in some Asteraceae is not simultaneous and instead follows a successive formation of the organs in the same whorl, in bidirectional order, as described in *S. vernalis* [45] and *X. squarrosum* [40].

The morphological evaluations of plants in the greenhouse showed that the first bud appeared externally in the center of the plants, on average, at 32.2 DAG, and anthesis was observed at 49.2 DAG. Thus, anthesis occurred on average 17 days following the first external appearance of the bud. This is consistent with observations in the common dandelion (*Taraxacum officinale*), where this period ranged from 12.5 to 18.9 days [50]. However, *T. officinale* took longer to show the first inflorescence bud externally, varying from 88.9 to 96 days after being transplanted to the greenhouse, i.e., 102.9 to 110 days after planting [50]. These differences could be explained in part by differences in the reproductive biology of the analyzed species (i.e., *T. officinale* being an apomictic polyploid and TK being a sexual self-incompatible diploid), despite them being in the same genus. Some degree of individual variation in flowering time was observed. This may be due to the genetic control of flowering in TK [31], enhanced by the extent of heterozygosity currently exhibited in TK germplasm, regardless of the use of a maternal half-sib family. Based on the evidence gathered in this study, flowering time segregation exists in the current TK breeding germplasm and should be further analyzed. More robust conclusions may be obtained by starting from a series of homogeneous genotypes showing distinct flowering times, including extremely early and biennial phenotypes. In addition, the understanding of the relationship of flower-development traits with rubber production and concentration in the roots is fundamental for the development of TK as a crop. TK plants flowering early in the first year showed lower rubber content, and their seeds generated a lower rubber-content population (Whaley et al., 1947). Moreover, it has been seen that some plants developed flowers in the first year while others did not, with the latter showing larger roots, higher productivity, and higher rubber content (Whaley et al., 1947).

The floral development of some genera in the Asteraceae family have been characterized. However, as far as we know, this study represents the first description of the *Taraxacum* genus using scanning electron microscopy. The results presented here will enable the design of studies focusing on the determination of the best floral developmental stages for the collection of ovules and pollen grains explants for somatic embryogenesis induction in vitro, and thus the production of haploid plants. Molecular studies can also be developed to understand the regulatory mechanisms in gene expression during flowering and inflorescence development in TK and related diploid sexual species. The understanding of floral development also has relevant implications for the determination of appropriate cultural practices of agronomic management (e.g., fertilization and pesticide application), for the production of the rubber cropping system, and for commercial seed production. Therefore, the characterization of floral development in TK is of crucial importance for establishing this new crop in large scale production and industrial supply.

## 4. Materials and Methods

### 4.1. Plant Material

Seeds from the maternal half-sibling family 305-05 of TK were used for this study. A total of 546 plants were grown and maintained in the greenhouse at the Ohio Agricultural Research and Development Center of The Ohio State University in Wooster, OH, USA, from September 2017 to January 2018. Seeds were sown in individual cones (20 × 4 cm) containing PRO-MIX^®^ (Premier Tech Horticulture, Montgomery, AL, USA) growing medium and were cultivated with supplemental lighting.

### 4.2. Morphological Evaluation

Three hundred and sixty plants from three planting dates (15 September, 29 September, and 15 October 2017) were collected for morphological and microscopy analysis, at every 3- or 5-day intervals after germination. Stages of development were recorded based on days after germination (DAG): observed at 5, 10, 15, 18, 21, 24, 27, 30, 33, 36, 39, 42, 45, and 48 DAG. For sampling, 26, 30, 30, 21, 31, 31, 28, 28, 28, 29, 23, 27, 13 and 16 plants were sampled, respectively. These plants were evaluated based on the total number of true leaves in the plant, length of the longest leaf (cm), and number of externally visible bud inflorescences (in the center of the plant rosette) at the time of collection. Pictures of the whole plants were taken in the greenhouse using a Canon EOS Rebel T6i 750D camera with a Canon 50 mm EF lens (Canon Inc., Melville, NY, USA).

### 4.3. Inflorescence Development and Floral Organogenesis

The developmental process of inflorescence and floral organogenesis were observed under a stereomicroscope and a scanning electron microscope. The selected plants described above (collected at 5, 10, 15, 18, 21, 24, 27, 30, and 33 DAG) were dissected using a Leica S6D stereomicroscope and fixed in FAA (37% formaldehyde/glacial acetic acid/95% ethanol/dH_2_O, 10:5:50:35, *v*/*v*) at least over night at room temperature [51]. Samples were then dehydrated for 15 min in each of the following concentrations 50%, 70%, and 90% ethanol (1×/ea.) and 100% ethanol (3×) at room temperature [34]. Critical point drying was obtained (Samdri-790, Tousimis Research Corporation, Rockville, MD, USA) mounted on aluminum stubs and platinum coated. One hundred and five plants were sampled, and their inflorescences observed and imaged using scanning electron microscopy (Hitachi S-3500N, Tokyo, Japan) under high vacuum.

To evaluate the timing of inflorescence development and floral organogenesis, a total of 98 plants were evaluated considering the developmental stage of the oldest inflorescence in the plant. The developmental stage was assigned according to the nine stages proposed herein for inflorescence development in TK.

### 4.4. Timing of Inflorescence Bud Appearance and Anthesis

The visual analysis of 58 plants was conducted in the greenhouse considering “time to bud” (in DAG), which represents the day when the first inflorescence bud was visible in the center of the rosette of leaves. Forty-four plants were evaluated for “time to anthesis” (when the inflorescence was completely opened). The second (*n* = 35 plants sampled), third (*n* = 23), and fourth (*n* = 19) inflorescences were recorded when they appeared in the center of the plant. Plants were evaluated every 1, 2, or 3 days from the date of planting until 60 DAG.

## 5. Conclusions

The present study shows a description of inflorescence and floral development in TK, proposing nine stages of inflorescence development using SEM techniques. The study showed that flowers were formed in an acropetal order in the capitulum. In relation to floral development, the first formed organ whorl was the corolla ring, followed by the androecium, pappus primordia, and finally, the gynoecium. During the initiation of whorls, organs in the same whorl developed simultaneously. This study also showed that the pappus primordium in TK was initiated forming a ring around the flower. The inflorescence stages were correlated to the morphological description of the flowers, and the first conversion of the vegetative meristem to a reproductive meristem was noticed at 21 DAG in plants showing, on average, 7.2, the longest leaf measuring 4.0 cm, and 0.1 buds appearing externally in the center of the plant. On average, the first floral bud appeared externally in the center of the plants at 32.2 DAG, while anthesis was noticed at 49.2 DAG. The work presented here represents basic knowledge of fundamental interest for the neo-domestication of TK, concerning flowering, pollination, seed production, and rubber accumulation. Furthermore, it is expected that the results presented here can yield information for further experiments and deeper knowledge regarding TK development, physiology, nutrition, morphology, biotechnology, plant breeding, crop management, and the development of agricultural practices. This research can contribute to enhance the knowledge related to this important natural rubber- and latex-producing alternative crop.

## Supplementary Materials

The following are available online at https://www.mdpi.com/2223-7747/9/10/1258/s1, Figure S1: Inflorescence stages (from A to I) observed 5 to 33 days after germination (DAG) in *Taraxacum kok-saghyz*, Figure S2: Development of *Taraxacum kok-saghyz* in the greenhouse showing the stages and dates they were collected (in days after germination; DAG), Figure S3: Date of 1st, 2nd, 3rd, and 4th inflorescence bud visually apparent and date of anthesis in days after germination (DAG) in *Taraxacum kok-saghyz*; *n* = 19–47. Bars represent the standard deviation (SD).
