# Supplementary material for: Inflorescence Development and Floral Organogenesis in Taraxacum kok-saghyz
Source: Plants (Basel). 2020 Sep 24;9(10):1258. doi: 10.3390/plants9101258 (PMC7650721; doi:10.3390/plants9101258)
Supplement: Supplementary file 1 [file plants-09-01258-s001.zip › plants-915780_supplementary/plants-supplementary.pdf]

Supplemental figures

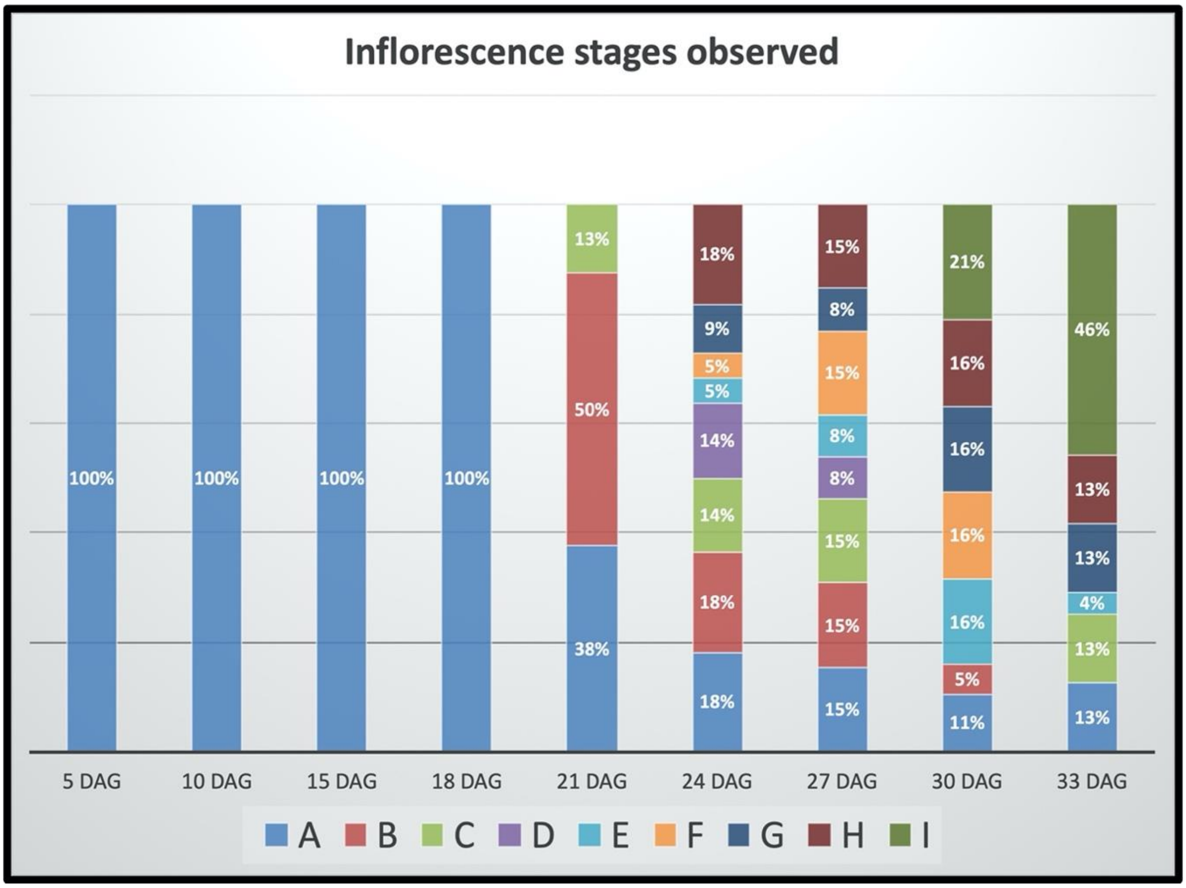

Figure S1. Inflorescence stages (from **A** to **I**) observed 5 to 33 days after germination (DAG) in *Taraxacum kok-saghyz*.

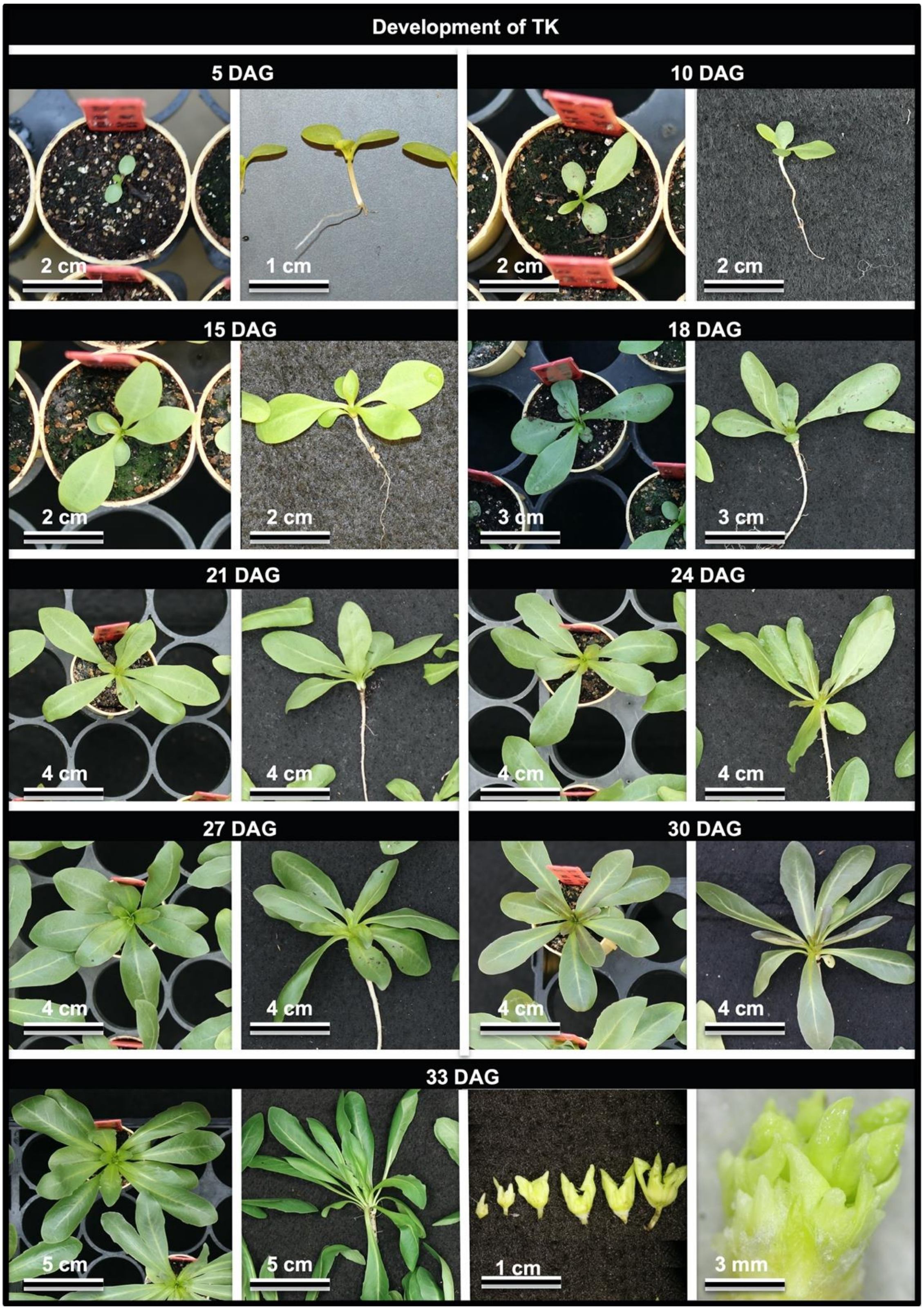

Figure S2. Development of *Taraxacum kok-saghyz* in the greenhouse showing the stages and dates they were collected (in days after germination; DAG).

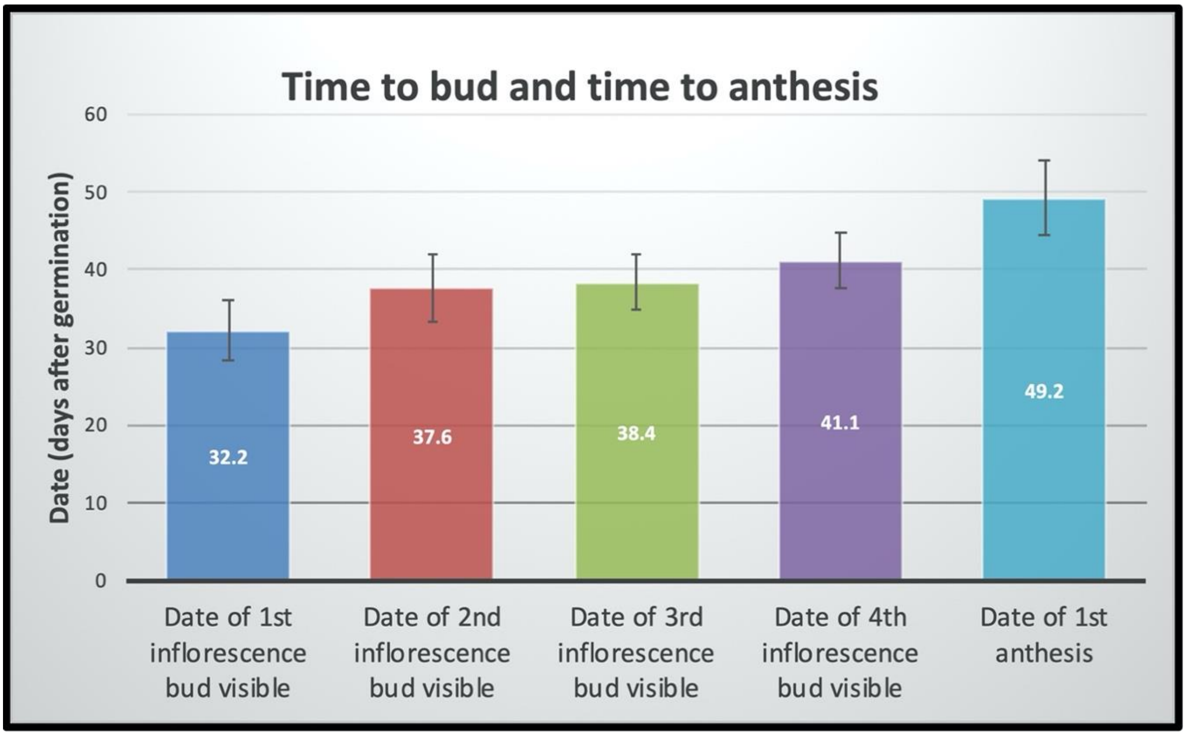

Figure S3. Date of 1st, 2nd, 3rd, and 4th inflorescence bud visually apparent and date of anthesis in days after germination (DAG) in *Taraxacum kok-saghyz*. Biological replicates, n = 19-47. Bars represent the standard deviation (SD).
