# Supplementary figures and images for: Inflorescence Development and Floral Organogenesis in Taraxacum kok-saghyz
Source: Plants (Basel). 2020 Sep 24;9(10):1258. doi: 10.3390/plants9101258 (PMC7650721; doi:10.3390/plants9101258)

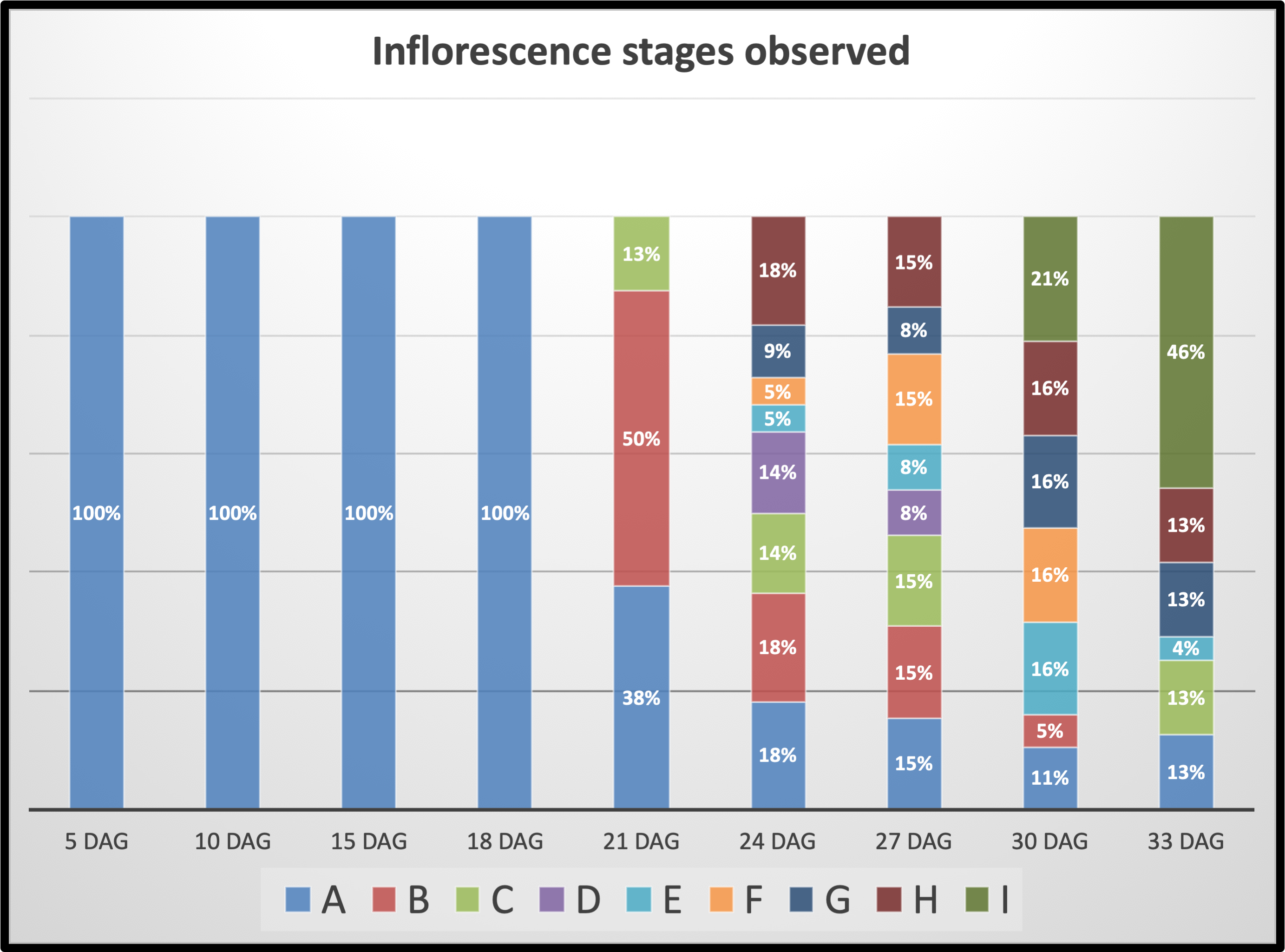

Supplement: Supplementary file 1 [file plants-09-01258-s001.zip › plants-915780_supplementary/Supplemental figure 1.tiff]

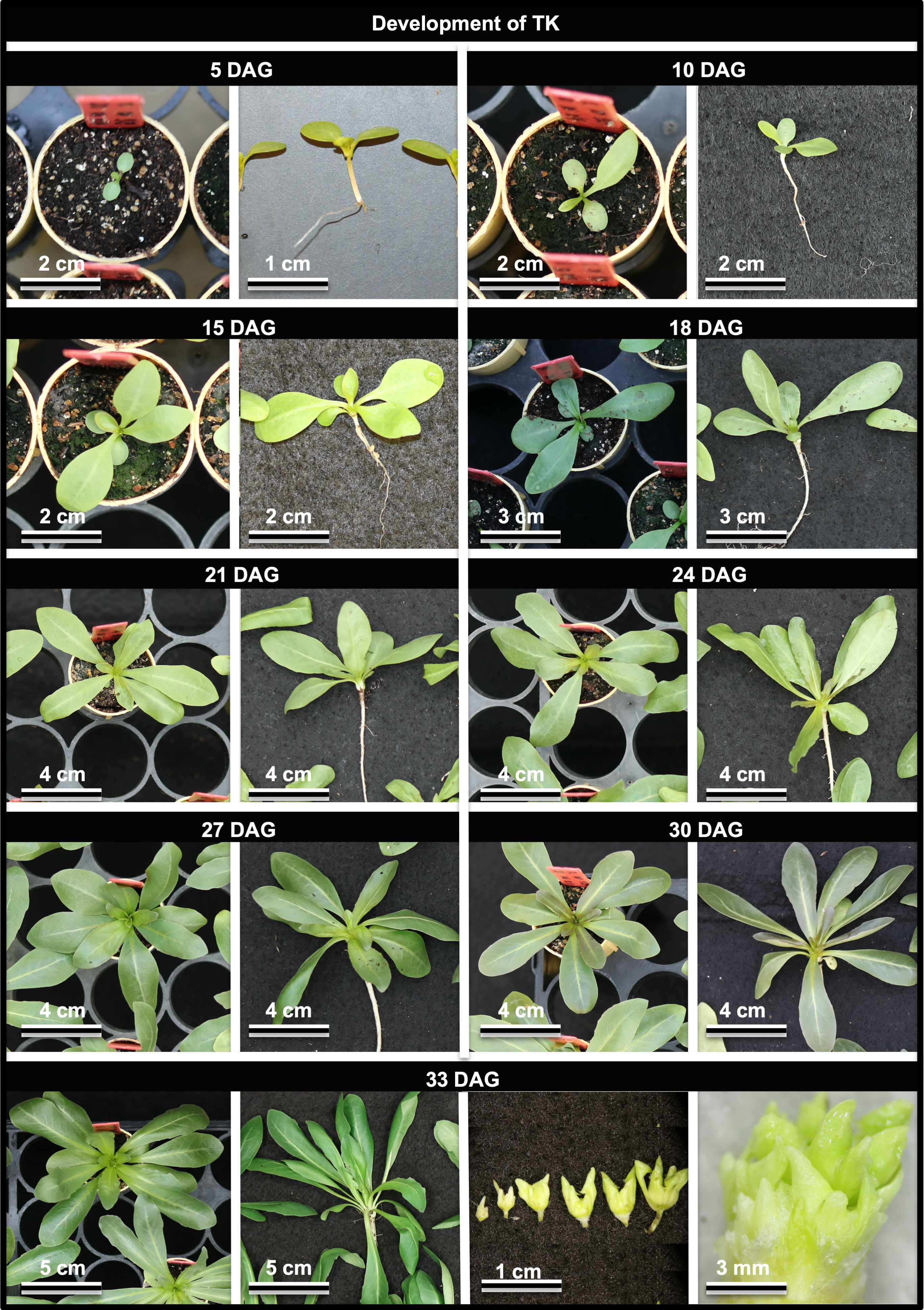

Supplement: Supplementary file 1 [file plants-09-01258-s001.zip › plants-915780_supplementary/Supplemental figure 2.tiff]

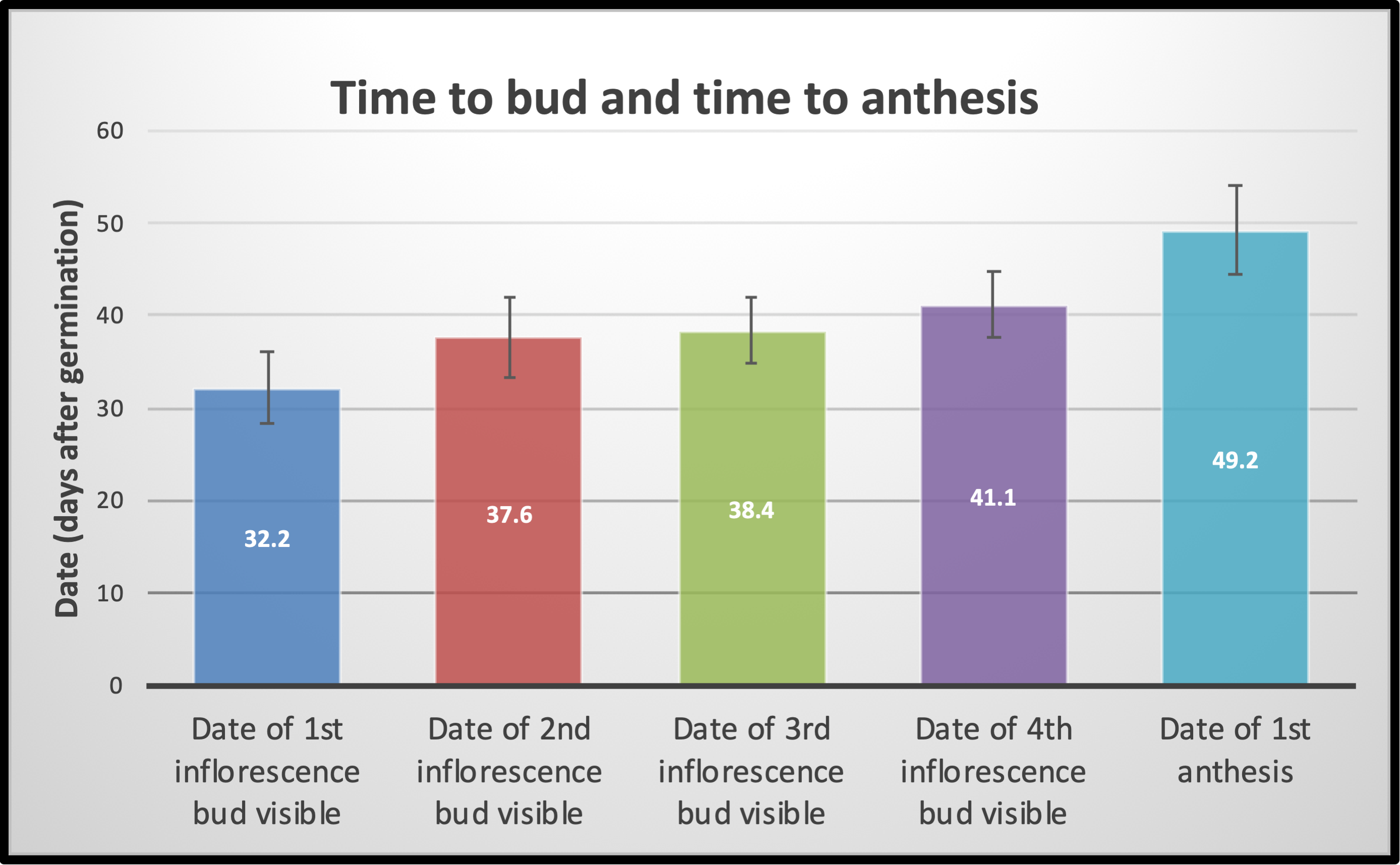

Supplement: Supplementary file 1 [file plants-09-01258-s001.zip › plants-915780_supplementary/Supplemental figure 3.tiff]
